# Gall-induction in insects: evolutionary dead-end or speciation driver?

**DOI:** 10.1186/1471-2148-10-257

**Published:** 2010-08-25

**Authors:** Nate B Hardy, Lyn G Cook

**Affiliations:** 1 Queensland Primary Industries and Fisheries, Entomology, Brisbane, Queensland 4068, Australia; 2 The University of Queensland, School of Biological Sciences, Brisbane, Queensland 4072, Australia

## Abstract

**Background:**

The tree of life is significantly asymmetrical - a result of differential speciation and extinction - but general causes of such asymmetry are unclear. Differences in niche partitioning are thought to be one possible general explanation. Ecological specialization might lead to increases in diversification rate or, alternatively, specialization might limit the evolutionary potential of specialist lineages and increase their extinction risk. Here we compare the diversification rates of gall-inducing and non-galling insect lineages. Compared with other insect herbivores feeding on the same host plant, gall-inducing insects feed on plant tissue that is more nutritious and less defended, and they do so in a favorable microhabitat that may also provide some protection from natural enemies. We use sister-taxon comparisons to test whether gall-inducing lineages are more host-specific than non-galling lineages, and more or less diverse than non-gallers. We evaluate the significance of diversity bipartitions under Equal Rates Markov models, and use maximum likelihood model-fitting to test for shifts in diversification rates.

**Results:**

We find that, although gall-inducing insect groups are more host-specific than their non-galling relatives, there is no general significant increase in diversification rate in gallers. However, gallers are found at both extremes - two gall-inducing lineages are exceptionally diverse (Euurina sawflies on Salicaceae and *Apiomorpha *scale insects on *Eucalytpus*), and one gall-inducing lineage is exceptionally species-poor (*Maskellia *armored scales on *Eucalyptus*).

**Conclusions:**

The effect of ecological specialization on diversification rates is complex in the case of gall-inducing insects, but host range may be an important factor. When a gall-inducing lineage has a host range approximate to that of its non-galling sister, the gallers are more diverse. When the non-galler clade has a much wider host range than the galler, the non-galler is also much more diverse. There are also lineage-specific effects, with gallers on the same host group exhibiting very different diversities. No single general model explains the observed pattern.

## Background

**Figure 1:**
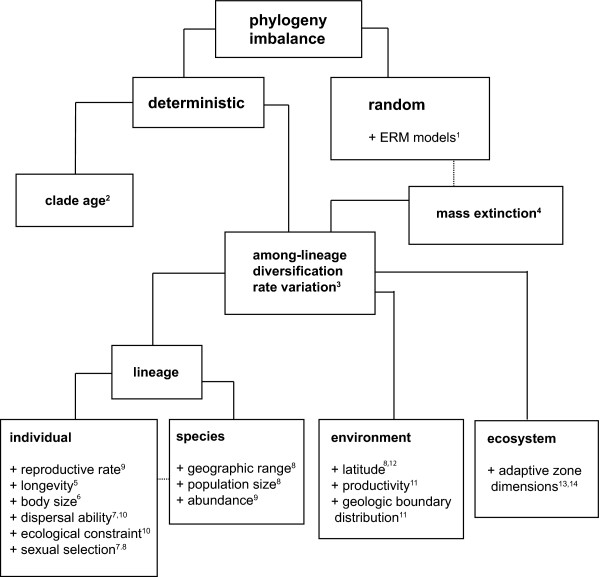
**Classification of causes of tree imbalance**. Examples of attributes that are commonly discussed in the literature are preceded by '+,' with references in superscript. Mass extinction is shown as both random and deterministic; mass extinctions are stochastic events, but not the type captured by purely neutral ERM models.

The tree of life is significantly less balanced than expected under a stochastic process of lineage divergence and extinction [1] - some lineages are diverse whereas others are species-poor. Deterministic explanations for the asymmetry include clade age [2], and among-lineage diversification rate variation [3] caused by mass extinction [4], lineage attributes [5-9], environmental attributes [10,11], and ecosystem attributes [12,13] (Figure 1). Lineage attributes affecting diversification rates can be divided into two classes: (1) phenotypic traits that are attributes of individuals, for example reproductive rate, dispersal ability, and the degree of ecological specialization; and (2) traits that are attributes of species, for example geographic range, population size, and local abundance. A key factor in the theory of diversification rate variation is resource availability and breadth, i.e. adaptive zone dimensions. Under an adaptive radiation model [14-16] it is argued that diversification is limited to the amount of free space in an adaptive zone, and that elevated rates of diversification are driven by ecological opportunities in geographic space (e.g. island colonization) or the evolution of an adaptive trait (key innovation). Well-studied examples of adaptive radiation include the Hawaiian silverswords [17], phytophagous beetles [12,18], and columbines [16]. Ecological specialization is thought to be an important process following expansion of a lineage's adaptive zone, and a major driving force generating species richness and diversity [15,19-21].

Not all adaptive traits are expected to result in an increased rate of net diversification; on the contrary, some adaptive traits may result in a dramatic depression of the diversification rate e.g. in-bred sociality in theridiid spiders [22]. Reduced radiation rate as a result of failure to speciate is commonly interpreted to result in "evolutionary dead-ends" - lineages that have low adaptation potential and are thought likely to become extinct before they can diversify [23,24]. Thus, the expected effect of ecological specialization on diversification rate has been an area of debate [25]. On the one hand, diversification rate is likely to increase if specialists have smaller geographic ranges and population sizes [26]. On the other hand, tightly constrained niches are likely to be unstable over time, and it has been predicted that specialization should be associated with increased extinction rates due to a specialist's inability to adapt [23,27,28], a notion supported by some empirical analyses [29,30].

The evolution of gall-induction on plants is a major trophic shift that has occurred multiple times among insects, with over 13,000 described species with this habit [31]. Galls are believed to provide the inducer with enhanced nutrition, a favorable microclimate and, in some cases, protection from natural enemies [32]. Among nematine sawflies, gall-inducing species are targeted by fewer species of parasitoid and experience lower rates of mortality than external-feeding species [33], but in general natural enemies can inflict high mortalities on gallers, and top-down selection pressure is thought to be a critical force driving interspecific variation in gall form [32,34,35]. Insect galls are thus an extended phenotype of their inducers - with the gall exposed to selection pressures related to predation and host resistance [32].

Gall-inducing taxa appear to be exceptionally host- and tissue-specific. For example, less than 1% of the described species of gall wasps (Cynipidae) have been recorded from more than one host genus [36], and gall wasps on *Quercus *are the single most diverse lineage of herbivores associated with a single host genus (about 1000 described species [36]). All of the 640 described species of agaonid fig wasps induce galls within the flowers of species of *Ficus *[37]. Within-host-plant diversification has occurred in many groups of gall midges [38,39] with, for example, a monophyletic group of 14 species of *Asphondylia *(Diptera: Cecidomyiidae) inducing galls on the leaves, stems, buds and flowers of a single plant species (*Larrea tridentate*) [40]. Only three of the fourteen described species of the gall-inducing psyllid genus *Calophya *(Psylloidea: Calophyidae) associated with *Schinus *(Anacardiaceae) are known to develop on more than one host species - there are eight species of *Schinus *[41]. Gall-inducing species of aphids and scale insects also tend to be constrained to closely related hosts [42-45].

These observations of host-specificity, and the intimate relationship between gall-inducer and host, have led to the idea that gall-inducers are specialized relative to their externally-feeding progenitors [46,47]. Gall-inducing insects thus provide a good study system to examine the effects of ecological specialization on evolutionary diversification rates.

Here, we examine phylogenies of gall-inducing insects and their non-galling relatives, including a total of approximately 1,650 species (Additional files 1,2,3,4). We first use thirteen sister-taxon comparisons to test the assumption that gallers are more host-specific than non-gallers. We then evaluate the significance of any diversification asymmetry against an Equal Rates Markov (ERM) model. Finally, we use maximum likelihood to compare the fit of fixed (1 speciation rate) and flexible (2 or more speciation rates) birth-death models to phylogenies, to test whether inferred origins of gall induction correlate with a shift in diversification rate [48,49].

## Results

### Host-specificity

Gall-inducing insects are significantly more host-specific than their non-galling sister groups (exact one-tailed Wilcoxon signed-rank test p-value = 0.050). All five significant differences in host range favored gallers being more host specific than their non-galling sister group.

### Diversification rates

#### Sister-taxon comparisons

The gall-inducing sister-clade was more diverse in seven of thirteen comparisons, and the non-galling sister was more diverse in the other six. We cannot reject the null model in which each sister has an equal chance of being more diverse with the binomial test (exact two-tailed test p-value = 1) or the signed-rank test (two-tailed p-value = 0.95).

One gall-inducing taxon was more diverse than expected under an Equal Rates Markov (ERM) null model: the scale insect genus *Apiomorpha *(p-value = 0.020). Two gall-inducing taxa were more diverse with marginal non-significance: Cerataphidini aphids (p-value of 0.053), sawflies in the subtribe Euurina (p-value = 0.059). Two gall-inducing taxa were markedly less diverse than expected: the armored scale insect genus *Maskellia *(p-value = 0.0023) and the sawfly genus *Micronematus *(p-value = 0.033).

#### ML (birth-death) modeling of shifts in radiation rate

Only two data sets were sufficiently sampled to satisfy criteria for tests of rate shifts along lineages. For the *Acacia *thrips, we were unable to reject the fixed-rate null model. In the LASER analysis, under no extinction (ϵ = 0), the likelihood ratio test (LRT) p-value was 0.096. Under high rates of extinction (ϵ = 0.95) the LRT p-value was 0.17. The MEDUSA analysis selected a one-rate model with a net diversification rate of 0.042 and a relative extinction rate of 0.13. All more complicated models had higher Akaike information criterion (AIC) scores.

For the nematine sawflies, the LASER analysis strongly favored the flexible-rate model (LRT p-value << 0.0001 under no extinction and high extinction) with an increase in diversification rate along the stem branch of the Salicaceae-galling Euurina. The MEDUSA analysis selected a model with 4 shift points. As in the LASER analysis, the single shift point resulting in the largest increase in likelihood was at the stem node of the Euurina. Background rates of net diversification (*r*) and relative extinction (ϵ) were estimated as 0.048 and 0.69 respectively. Within Euurina both the net diversification rate and relative extinction rate increased dramatically (*r*= 0.094, ϵ = 0.99).

## Discussion

We confirm that, as expected and commonly stated, gall-inducing taxa are more host-specific than their non-galling relatives. The effect of ecological specialization in gall-inducing insects on diversification rates is complex, but host range appears to be a critical factor. When a gall-inducing lineage has a host range approximate to that of the non-galling sister, the gallers are more diverse. These include the Salicaceae-galling Euurina gall wasps that are sister to a group restricted to Salicaceae and Betulaceae, the *Styrax*-galling aphids Cerataphidini that are sister to a group restricted to *Quercus*, and the *Eucalyptus*-galling scale insect genus *Apiomorpha *that is sister to a group that occurs on *Eucalyptus *and Casuarinaceae. Conversely, when the non-galling clade has a much wider host range than the galler, the non-galler is much more diverse. In our analysis, the diversity bipartition that is most significantly weighted in favor of the non-galling taxon (*Maskellia *sister to a taxonomically heterogeneous group of armored scales) is also the comparison with the greatest disparity in host range, as measured by the estimated age of the most recent common ancestor of the hosts. The importance of host breadth as a determinant of diversification rate is consistent with the assumed importance of host-switching in speciation of phytophagous and parasitic lineages [46,50].

Thus adaptive zone dimensions may be of more fundamental importance to diversification rate variation than is the degree of ecological specialization. When clade host breadth was roughly equivalent between gall-inducing and non-galling sister groups, the gall-inducing group was more diverse. This could result from uneven diversification rates stemming from differences in species-level ecological specificity, or because, for a given set of hosts, the adaptive space available to a gall-inducing lineage is larger than that presented to a non-galling species.

Ecological traits of host lineages are also likely to affect diversification rates of gall-inducers. Two of the diverse gall-inducing groups in this study, Euurina and *Apiomorpha*, occur on host taxa (*Salix *and *Eucalyptus *respectively) that are persistent and locally abundant over vast geographic spaces, traits thought to reduce a lineage's probability of extinction. On the other hand, the most strikingly species-poor gall-inducing taxon included in this study, *Maskellia*, also occurs on *Eucalyptus*, and the gall-inducing thrips on *Acacia *are not as diverse as might be expected given the diversity of the host (more than 1000 described species of *Acacia *[51]), although it likely that much of the true species diversity of gall-inducing thrips is unrecognized [52].

Gall-inducer diversification rates could also be profoundly affected by top-down pressure from parasitoids and pathogens. Gall-inducing Euurina sawflies have been shown to have a depauperate fauna of natural enemies and suffer lower enemy-caused mortality than closely related external feeders. In contrast, species of *Apiomorpha *experience extremely high mortality rates (LGC personal observations).

## Conclusions

Gall-inducing lineages tend to be more host-specific than their non-galling sister groups. A gall almost certainly represents an expansion of the ecological resource pool (e.g. new tissues and, at least initially, enemy-free spaces), but the gall-inducer's niche space may not be expanded. The effect of the evolution of gall-induction on net diversification rate appears to have been lineage-specific; no general trend was detected, but some gall-inducers were exceptionally diverse and others were exceptionally under-diverse. Although this study was not designed explicitly to test the effect of a lineage's host-breadth on its net diversification, our results suggest that host-breadth may be a general factor influencing the net diversification of phytophagous insect groups.

## Methods

### Data

We surveyed the literature for published phylogenies containing gall-inducing taxa and their non-galling relatives. We also used the PhyLoTA Browser rel. 1.01 [53] to survey the nucleotide sequence data deposited in GenBank for groups containing gall-inducers. Our dataset included thirteen galler and non-galler sister clades (Table 1). Unless otherwise noted, extant diversity estimates were derived from the literature (see Additional file 1). We estimated phylogenies when: (1) a sister relationship of interest had not been inferred with support in a published analysis and/or additional DNA sequence data had subsequently become available; (2) phylogenies and extant diversity were deemed sufficient (we would be able to allocate all extant species diversity among terminal nodes) for ML diversification rate model fitting. Details of the phylogenetic datasets and results for individual taxa are provided as Additional Material.

**Table 1:** Comparisons between galling and non-galling insect sister groups

Galling-sister	Species number	Host range	Gall host MRCA age (Ma)	ERM P galler	Non-galling sister	Species number	Host range	Non-gall host MRCA age (Ma)	ERM P non-galler
Euurina	400	*Salix*, *Populus*	58	0.059	*Nematus melanaspis*-group	25	Salicaceae, Betulaceae	94	0.94
*Bacconematus*	1	*Ribes*	35	1.00	*Eitelius*	2	*Salix*	35	0.50
*Pristolina*	4	*Vaccinium*	0	0.50	*Pristicampus*	3	*Potentilla*	0	0.67
*Micronematus*	1	*Prunus*	70*	0.97	*Pristiphora *subgenus *Sala*	30	Fagaceae, Betulaceae, Salicaceae, Fabaceae, Rosaceae, Grossulariaceae, Malvaceae	114	0.033
*Kladothrips*	24	*Acacia*	0	0.21	*Rhopalothripoides*	6	*Acacia*	0	0.83
gall-inducing Cynipidae + Synergini	1369	Fagaceae, Rosaceae, Papaveraceae, Lamiaceae, Asteraceae, Anacardiaceae, Smilacaceae, Valerianaceae, Apiaceae, Sapindaceae	144	0.24	Figitidae (in part): Charpinae, Anacharitinae, Figitinae, Aspicerinae	435	Diptera, Neuroptera, Hymenoptera	300	0.76
Agaonidae + Otitesellinae	690	*Ficus*	60	0.15	Sycoryctinae	121	Agaonidae + Sycoryctinae	60	0.85
Eurostina	41	Asteraceae (Soldagininae: *Solidago*; *Chrysothamnus*; *Gutierrezia*))	0	0.54	Euaresta	46	Asteraceae (Ambrosiinae: *Ambrosia*; *Xanthium*; *Dicoria*)	0	0.48
Oedapidina	88	Asteraceae	42	0.71	Tephrellini	210	Acanthaceae, Lamiaceae, Verbenaceae	45	0.30
*Hexomyza*	16	Liliopsida/Eudicotyledons	144	0.95	*Ophiomyia *+ *Tropicomyia*	282	Liliopsida/Eudicotyledons	144	0.054
*Apiomorpha*	150	*Eucalyptus*	65	0.020	*Ourococcus*	3	Myrtaceae, Casuarinaceae	104	0.99
*Maskellia*	2	*Eucalyptus*	65	1.00	Aspidiotini; Pseudaonidina; Odonaspidini; non-pupillarial Parlatorini	874	Magnoliphyta/Coniferophyta	366	0.0023
Cerataphidini	73	*Styrax*	0	0.053	Thelaxes (Thelaxinae)	4	*Quercus*	0	0.96

Most recent common ancestor (MRCA) ages are in millions of years before the present. Host ranges are reported as lists of taxa or, in the case of diverse ranges, as two subgroups which span the MRCA node. Divergence dates are from Davies et al. [69] (angiosperms) and Hedges et al. [70]

### Phylogenetics

Unaligned FASTA files were downloaded from PhyLoTA, and aligned using MUSCLE v.3.6 [54]. Ribosomal alignments were filtered through the Gblocks server [55,56], with each of the options for less stringent selection chosen, to remove areas of high alignment ambiguity. Introns were excluded from nuclear protein-coding loci. If multiple loci were available, these were concatenated. Datasets were partitioned by genome, and by codon position for protein-coding loci. Maximum likelihood (ML) trees were inferred using RAxML v.7.0.3 [57], with the parameters of a general time reversible (GTR) nucleotide substitution model estimated independently for each data partition. Among-site rate variation was estimated under CAT approximation during 100 nonparametric bootstrap pseudoreplicates. Every fifth bootstrap tree was then used as a starting tree for more thorough ML optimization with gamma-distributed rate variation.

### Host specificity analysis

In order to remove taxonomic bias from measures of host breadth, host range was measured as the age (Ma) of the most recent common ancestor (MRCA) of the hosts. This approach to quantifying host breadth is akin to phylogenetic diversity (PD) [58], the minimum total length of branches that span a given set of taxa on a phylogenetic tree. Our metric is distinct, however, in that branch lengths have been scaled to time rather than raw branch length. Because age is standard across analyses, it could be used to make comparisons across DNA sequence datasets with variable substitution rates. It is a measure of the evolutionary depth of host breadth, whereas PD also accounts for the phylogenetic density of host use. None of the sister taxa used in our comparisons was restricted to a single host species.

For each of 13 pairs of gall-inducing and non-galling insect sister pairs, we recorded the host range of each sister. In cases for which each sister in a comparison was restricted to a single host genus or family, and our knowledge of host phylogeny and/or insect host breadth was insufficient to identify an age for the MRCA, we assumed there was no difference in host range. We used the Wilcoxon Signed-Rank Test to assess if the observed disparity in host breadth departed significantly from that expected under a null model in which each sister has an equal chance of having a broader host range. The test was one-tailed, reflecting our prior expectation, derived from the literature, that gall-inducing taxa would be more host-specific.

### Diversification rate analyses

#### Sister-taxon comparison

Some tree imbalance is expected under null models of stochastic diversification [59], and this needs to be taken into account in comparison of diversification rates. We compared the extant diversity of 13 monophyletic groups of gall-inducing insect species to the extant diversity of their sister taxa, against a null model in which the extant diversity of each sister has a 0.5 probability of being larger [7], and evaluated significance using the binomial and signed-rank tests, contrasting species richness with log-transformed ratios (as in [60], and recommended in [61]).

For each individual diversity bipartition we also calculated equal rates Markov (ERM) probabilities (using the equation 3 of Slowinski and Guyer [62]) for alternative hypotheses in which gall-inducing taxa were expected to more or less diverse, with a Bonferroni correction for multiple comparisons (α/2 = 0.025) to evaluate the significance of the departure from the null model. We did not follow the Slowinski-Guyer method of using Fisher's combined probability test to test the influence of a trait on diversification rates, because of the problems with that approach summarized by Vamosi and Vamosi [61].

#### ML tests for shifts in radiation rates

Optimal phylogenies were made ultrametric with nonparametric rate smoothing using r8 s v.1.70 [63]. As only the relative node heights were needed, an arbitrary root height of 100 was fixed for each tree. We used the modifications of the ML birth-death model fitting methods of Magallon and Sanderson [48] implemented in the R package LASER [64]. The likelihood was calculated by comparing the observed species diversity of a clade to an expected species diversity given a stem group age and a net diversification rate (speciation rate - extinction rate) estimated from the whole tree. To test for shifts in diversification rate, a fixed null model, in which a single diversification rate was estimated for all lineages, was compared to a flexible alternative model in which an ancestral diversification rate is permitted to shift to a descendent rate along some branch in the tree. The likelihood calculations were repeated for shifts along each branch of the tree. Significance of the model comparison was determined on the basis of likelihood ratio tests (LRT). To ensure that our inferences were robust over a range of extinction rates, analyses were repeated under two values for the ratio of the extinction rate to the speciation rate: 0 (no extinction) and 0.95 (very high extinction rates).

We also sought shifts in diversification rates using a stepwise birth-death model fitting approach based on the AIC implemented as MEDUSA in the R package GEIGER [65,66]. The estimateExtinction and cutAtStem parameters were set to True, and a cutoff of 4 units was selected for the improvement in AIC score required to accept a more complex model.

### Automation

We provide a Python program, Systers (Additional file 5) to automate sister-taxon comparisons as outlined in Vamosi and Vamosi 2005 [62]. Species diversities of each sister clade are contrasted using raw differences [13], proportional differences [67], and log ratio differences [60]. Statistical significance is assessed in one of three ways, depending on the number of comparisons. For analyses with ≤ 10 contrasts, significance is assessed with a randomization test for matched pairs [68]. In brief, the sign of the contrast scores are permuted and the sum of the contrasts is found for each possible permutation. The fraction of possible sums more extreme than the observed sum is returned as a two-tailed p-value. For analyses with 11-19 contrasts, significance is assessed with a Wilcoxon signed-rank test, and for analyses with ≥ 20 contrasts significance is assessed by normal approximation of the Wilcoxon signed rank test.

## List of abbreviations

AIC: Akaike information criterion; ERM: equal rates markov; GTR: general time reversible; LRT: likelihood ratio test; ML: maximum likelihood; MRCA: most recent common ancestor; PD: phylogenetic diversity.

## Authors' contributions

NBH and LGC cooperated in the conception and design of the study as well as the drafting of the manuscript. NBH carried out the data-mining and analyses and wrote the Python program Systers. Each read and approved the final manuscript.

## Supplementary Material

Additional file 1 **Phylogenetic datasets and results**. Survey of DNA sequence-based phylogenetic studies including gall-inducing groups, and details of phylogenetic estimates performed here. Click here for file

Additional file 2 **ML tree estimated from aphid DNA sequence data**. Aphidoidea ML phylogeny estimated from analysis of EF1α, long-wavelength opsin, COI, COII, cytochrome b, NADH dehydrogenase 1, ATP synthase subunit 6, and mitochondrial ribosomal subunits 12 S and 16 S dataset partitioned by genome and codon position. Major lineages are labeled following the classification used by Blackman and Eastop. Click here for file

Additional file 3 **ML tree estimated from cynipoid DNA sequence data**. Cynipoidea ML phylogeny estimated from analysis of 28 S, 18 S, and COI dataset partitioned by genome and codon position. Deep relationships supported by >70% bootstrap proportions denoted by stars. Empty circle indicated clade of unpublished Cynipini sequences assumed to be misidentifications of synergine inquilines. Click here for file

Additional file 4 **ML tree estimated form chalcidoid DNA sequence data**. Chalcidoidea ML phylogeny estimated from analysis of 28 S sequences. Deep relationships supported by >70% bootstrap proportions denoted by stars. Group A composed of exemplars of the following families: Aphelinidae, Chalcididae, Encyrtidae, Eucharitidae, Leucospidae, Mymaridae, Pteromalidae, Perilampidae, Tetracampidae; Group B composed of exemplars of: Aphelinidae, Eurytomidae, Ormyridae, Perilampidae, Pteromalidae, Tanaostigmatidae, Tetracampidae, Torymidae. Click here for file

Additional file 5 **Python script to automate sister taxon comparisons**. A program that reads sister clade species diversities in a CSV file, calculates diversity contrasts with multiple metrics, and evaluates significance with either Siegel's randomization test for matched pairs, the Wilcoxon signed rank test, or normal approximation of the Wilcoxon signed rank test. Click here for file
