# Supplementary material for: Gall-induction in insects: evolutionary dead-end or speciation driver?
Source: BMC Evol Biol. 2010 Aug 25;10:257. doi: 10.1186/1471-2148-10-257 (PMC2939573; doi:10.1186/1471-2148-10-257)
Supplement: Additional file 1 — Phylogenetic datasets and results. Survey of DNA sequence-based phylogenetic studies including gall-inducing groups, and details of phylogenetic estimates performed here. [file 1471-2148-10-257-S1.DOC]

**Phylogenetic datasets and results.**

*Sawflies*

Among the nematine sawflies (Tenthredinidae) are gall-inducing lineages associated with Salicaceae (Euurina), *Ribes* (*Bacconematus*), *Vaccinium* (*Pristolina*) and *Prunus* (*Micronematus*) [1]. Nyman et al. [2] inferred a well-supported phylogeny of the nematine sawlfies based on sequences of elongation factor-1 alpha (EF1α) and cytochrome oxidase subunit I (COI) (81 taxa; 1587 sites). They provided their concatenated alignment as supplemental material that we downloaded from the *Systematic Entomology* website. For the ML birth-death model fitting, we excluded all taxa outside the Nematinae (because of uncertainty about the extant diversity associated with those branches) and pruned all but one lineage per clade of known diversity (as in [3]). In most cases this translated to one species per genus but, in the case of the diverse but paraphyletic genus *Nematus*, all but one taxon was pruned from each of the monophyletic species groups inferred by Nyman et al. [2]. The resulting tree contained a single polytomy that was resolved arbitrarily by adding a zero-length branch using Mesquite v. 2.72 [4]. The pruned sawfly dataset contained 44 taxa and 1587 sites.

*Acacia* thrips

More than 200 species of thrips are known from *Acacia* in Australia [5], including at least one gall-inducing lineage. We concatenated alignments of *Acacia* thrips EF1α, wingless, COI and the mitochondrial ribosomal subunit 16S [5,6]. The intron in the EF1α fragment was removed. All but one lineage per genus were pruned for ML birth-death model fitting. Our ML analysis of divergence rate variation requires an input tree that accounts for all of the extant diversity of the target group. DNA sequence data were unavailable for the *Acacia* thrips genera *Akainothrips* (33 described species), *Csirothrips* (21 sp.), *Katothrips* (35 spp.), and *Kellyia* (13 spp.), all of which are ‘opportunistic’ thrips that shelter within the abandoned galls and domiciles of other *Acacia* thrips species [5]. Without these taxa represented, our inferences should be considered provisional. The dimensions of the pruned *Acacia* thrips dataset are 19 taxa by 1969 sites.

McLeish et al. [7,8] used phylogenetic analyses of DNA sequence data to argue that *Kladothrips* diversification was driven by the diversification of host *Acacia* species brought about by Quaternary aridification of Australia. They included 29 undescribed *Kladothrips* taxa in their analyses, but did not explicitly delimit species. Here, we count only described species.

*Aphids*

Approximately 400 described species of aphids induce galls [9]. We estimated relationships among 611 aphid species with GenBank sequences from EF1α, long-wavelength opsin, COI, cytochrome oxidase subunit 2 (COII), cytochrome b, NADH dehydrogenase 1, ATP synthase subunit 6, and mitochondrial ribosomal subunits 12S and 16S [10,11,12,13,14,15,16,17,18,19,20,21,22,23,24,25, 26]. The estimated tree (Additional file 2) had low bootstrap support (BS) but in general the monophyly of morphologically-delimited groups was recovered. Two of the larger gall-inducing clades (Adelgidae + Phylloxeridae; Pemphigini + Fordini) could not be used in our host-specificity and diversification rates analyses because each had a sister group that contained a mixture of galling and non-galling species. The recovered non-monophyly of the Ulmaceae-galling Eriosomatini was at odds with strong morphological evidence [27, 28]; therefore, no sister relationship involving part of the Eriosomatini was considered. A sister relationship between the gall-inducing Cerataphidini and the non-galling Thelaxinae was used in our comparisons.

*Scale insects*

Cook and Gullan [29] used DNA sequence data (small nuclear ribosomal subunit 18S; EF1α; COI) to infer relationships among felt scales (Eriococcidae), the scale insect (Coccoidea) family with the highest number and proportion of gall-inducing species – approximately 100 / 500 of the described species. They recovered at least five origins of gall induction within the felt scales, with most of the species diversity in two clusters, (1) *Apiomorpha*, and (2) a clade comprised of *Lachnodius*, *Opisthoscelis* and several related genera.

Morse & Normark [30] inferred relationships among armored scale (Diaspididae) species from DNA sequence data from the loci EF1α and large ribosomal subunit 28S. Anderson et al. [31] refined this estimate with data from additional taxa and loci (COI and COII, as well as 16S sequences from the armored scale primary endosymbiont *Candidatus* Uzinure diaspidicola). Anderson et al. recovered support for a sister relationship between the species *Maskellia* *globosa* and a group containing most of the Aspidiotini, non-pupillarial Parlatorini, Pseudaonidina, and possibly the Odonaspidini. The genus *Maskellia* contains two described species, *M. globosa* and *M. nigra* [32], both of which are known to induce galls on the stems of *Eucalyptus*.

*Gall wasps*

The parasitic wasp family Figitidae was inferred to be sister to the gall wasps (Cynipidae) by parsimony analysis of morphological data [33], but in model-based analyses of DNA sequence (28S; 18S; and COI) and morphological data the monophyly of the Figitidae was not recovered [34]. Additional figitid and cynipid DNA sequence data were downloaded from GenBank [35,36, numerous unpublished sequences] and combined with those sequences used by Buffington et al. [34] for their inferences. After pruning redundant species and removing ambiguous regions in the alignment with GBlocks, our combined alignment consisted of the same three loci used by Buffington et al. [34] for 204 species and 2100 sites.

We failed to recover the monophyly of the Figitidae (Additional file 3), a result consistent with the model-based inferences of Buffington et al. [34] and the varying history of Figitidae classification [33]. The Cynipidae was monophyletic, with the exclusion of sequences labeled *Eschatocerus* *acaciae*, *Diplolepsis* *rosae,* *Pediaspis aceris* and *Liebelia* *fukudae*. The *D. rosae* and *L. fukudae* sequences were recovered without support as sister to a species of *Mytopsen* (Figitidae). The *E. acaciae* exemplar was sister to a species of *Thoreauella* (Figitidae) (low BS support, 41%). The *P. aceris* exemplar was sister to a clade containing *E. acaciae, D. rosae, P. aceris, L. fukudae,* and their sister groups, four Liopteridae species and a few additional figitids (*Aspericera* sp., *Paraspicera* sp., *Plectocynips* sp.)

In contrast to published phylogenies of the Cynipidae [33,36] the Synergini were inferred to be sister to the rest of the gall-wasps, although with only 47% BS support. Species in the Synergini are phytophagous inquilines capable of inducing the development of nutritive plant tissue inside galls of other cynipids [37]; thus, they are biologically more galler-like than non-gallers, and here we treat them as gallers. Five sequences attributed to gall-inducing species in the genera *Cynipis*, *Neutoterus* and *Trigonaspis* (Cynipini) were recovered as a clade deeply-nested within the Synergini. None of these sequences was included in a published study. Each of these genera was represented by additional species that were recovered within the Cynipini. Here, we assume that these sequences are from synergine inquilines mistakenly identified as the inducer of the gall from which they were collected. Because we have treated Synergini biology as gall-inducing, uncertainty associated with the relationship between the Synergini and other gall wasps should not bias our analyses.

The sister group of the Cynipidae was inferred (without BS support) to be a group of figitid taxa including exemplars from the subfamilies Anacharitinae, Aspicerinae, Charpinae, and Figitinae. We failed to recover monophyly for Aspicerinae; exemplars of the genera *Callaspidia* and *Omalaspis* were recovered within the Cynipidae sister group, but exemplars of *Aspicera* and *Paraspicera* were distantly related. Thus it is unclear what fraction of the known Aspicerinae species diversity (ca 100 species) should be allocated to the Cynipidae sister group. Given the non-directionality of our tests, and the knowledge that in this comparison the gall-inducing group contains 1369 species, the allocation that minimizes our chance of rejecting the null hypothesis is to allocate all of the un-sampled Aspicerinae diversity to the Cynipidae sister group.

*Fig wasps*

Relationships among the pollinating fig wasps (Agaonidae) and other chalcidoid taxa are unclear. To infer the sister group to the pollinating fig wasps, we analyzed an alignment of 444 chalcidoid 28S sequences [38,39,40,41,42,43,44,45,46, 47]. The matrix contained 500 sites after removing ambiguous region from the alignment with GBlocks. Family diversity estimates for the Chalcidoidea followed those given in the Universal Chalcidoidea Database (<http://www.nhm.ac.uk/research-curation/research/projects/chalcidoids/>), and FigWeb (<http://www.figweb.org/>).

The monophyly of the pollinating fig wasps (Agaonidae) was recovered with strong support (BS=0.86) (Additional file 4). The non-pollinating fig wasp subfamily Otitesellinae (Pteromalidae) was inferred to be the sister group of the agaonids, although without BS support. Sister to this group was a group of exemplars of the Sycoryctinae (Pteromalidae), parasitoids of gall-wasps. Monophyly of the Sycoryctinae was not recovered, Sycoscapter was sister to the rest of the Sycoryctinae + the fig-gall wasps, but there was little for these relationships.

Non-monophyly was recovered for several chalcidoid families: Aphelinidae, Eucharitidae, Myrmaridae, Perilampidae, Pteromalidae, Tetracampidae.

*Gall midges and gall flies*

Although the gall midge (Cecidomyiidae) supertribes Cecidomyiidi and Lasiopteridi, each with about 2000 described species, contain the most diverse gall-inducing lineages known [48], little is known about relationships within these groups and there are too few data available to analyse them here.

Gall fly (Tephritidae) relationships are also uncertain but a phylogeny of the subfamily Tephritinae based on 16S sequences [49] provided two comparisons: (1) Asteraceae-galling subtribe Eurostina sister to non-galling Asteraceae-feeding species in the subtribe Euaresta; and (2) Asteraceae-galling subtribe Oedapidina sister to species in the subtribe Tephrellini that develop on species of Acanthaceae, Lamiaceae and Verbenaceae. A few species classified within the Tephrellini have been reported to induce galls, although invariably these are singletons sister to non-galling species. Here we have treated the Tephrellini as non-galling.

*Leaf-mining flies*

The genus *Hexomyza* (Agromyzidae) is composed of fourteen described gall-inducing species. Scheffer et al. [50] used DNA sequence from COI, 28S, and the carbamoylphosphate synthase domain of CAD to estimate phylogenetic relationships among leaf-mining fly lineages. They recovered *Ophiomyia* + *Tropicomyia* as sister to *Hexomyza*. The sister group to Agromyzidae are Fergusoninidae [50]. Each of the approximately 20 species in the fly family Fergusoninidae is closely associated with nematodes that induce galls on myrtaceous hosts [51]. The diversity and host-specificity of fergusoninid flies were not compared to that of the leaf-mining flies because the latter group includes at least one gall-inducing lineage.

**References**

1. Nyman T, Zinovjev AG, Vikberg V, Farrell BD: Molecular phylogeny of the sawfly subfamily Nematinae (Hymenoptera: Tentredinidae). Systematic Entomology 2006, 31: 569-583.
2. Nyman T, Farrell BD, Zinovjev AG, Vikberg V: Larval habits, host-plant associations, and speciation in nematine sawflies (Hymenoptera: Tenthredinidae). Evolution 2006, 60: 1622-1637.
3. Rabosky DL, Donnellan SC, Talaba AL, Lovette IJ: Exceptional among-lineage variation in diversification rates during the radiation on Australia’s most diverse vertebrate clade. Proceedings of the Royal Society B 2007, 274: 2915-2923.
4. Maddison WP, Maddison SR: Mesquite: a modular system for evolutionary analysis. Version 2.72 http://mequiteproject.org
5. Morris DC, Schwarz MP, Crespi BJ, Cooper SJB: Phylogenetics of gall-inducing thrips on Australian *Acacia*. Biological Journal of the Linnean Society 2001, 74: 73-86.
6. Morris DC, Schwartz MP, Cooper SJB, Mound LA: Phylogenetics of Australian *Acacia* thrips: the evolution of behaviour and ecology. Molecular Phylogenetics and Evolution 2002, 25: 278-292.
7. McLeish MJ, Crespi BJ, Chapman TW, Schwarz MP: Parallel diversification of Australian gall-thrips on *Acacia*. Molecular Phylogenetics and Evolution 2007a, 43: 714-725.
8. McLeish MJ, Chapman TW, Schwartz MP: Host-driven diversification of gall-inducing *Acacia* thrips and the aridification of Australia. BMC Biology 5 2007b.
9. Blackman RL, Eastop VF: Aphids on the world’s trees. Oxford, UK: CAB International—Cambridge University Press; 1994.
10. Normark, B.B., 1999. Evolution in a putatively ancient asexual aphid lineage: recombination and rapid karyotype change. Evolution. 53: 1458–1469.
11. Normark, B.B., 2000. Molecular systematics and evolution of the aphid family Lachnidae. Molecular Phylogenetics and Evolution. 14, 131–140.
12. von Dohlen CD, Moran NA: Molecular data support a rapid radiation of aphids in the Cretaceous and multiple origins of host alternation. Biological Journal of the Linnean Society 2000, 71: 689-717.
13. Martinez-Torres D, Buades C, Latorre A, Moya A: Molecular systematics of Aphids and their primary endosymbionts. Molecular Phylogenetics and Evolution 2001, 20: 437-449.
14. Von Dohlen CD, Kurosu U, Aoki S: Phylogenetics and evolution of the eastern Asian—eastern North American disjunct tribe, Hormaphidini (Hemiptera: Aphididae). Molecular Phylogenetics and Evolution 2002, 23: 257-267.
15. Favret C, Voegtlin DJ: Speciation by host-switching in pinyon *Cinara* (Insecta: Hemiptera: Aphididae). Molecular Phylogenetics and Evolution 2004, 32: 139-151.
16. Inbar M, Wink M, Wool D: The evolution of host plant manipulation by insects: molecular and ecological evidence from gall-forming aphids on *Pistacia*. Molecular Phylogenetics and Evolution 2004, 32: 504-511.
17. Ortiz-Rivas B, Moya A, Martinez-Torres D: Molecular systematics of aphids (Homoptera: Aphididae): new insights from the long-wavelength opsin gene. Molecular Phylogenetics and Evolution 2004, 30: 24-37.
18. von Dohlen CD, Rowe CA, Heie OE: A test of morphological hypotheses for tribal and subtribal relationships of Aphidinae (Insecta: Hemiptera: Aphididae) using DNA sequences. Molecular Phylogeny and Evolution 2006, 38: 316-329.
19. Havill NP, Foottit RG, von Dohlen CD: Evolution of host specialization in the Adelgidea (Insecta: Hemiptera) inferred from molecular phylogenies. Molecular Phylogenetics and Evolution 2007, 44: 357-370.
20. Coeur d’acier A, Jousselin E, Martin JF, Rasplus JY: Phylogeny of the genus *Aphis* Linnaeus, 1758 (Homoptera: Aphididae) inferred from mitochondrial DNA sequences. Molecular Phylogenetics and Evolution 2007, 42: 598-611.
21. Pike N, Whitfield JA, Foster WA: Ecological correlates of sociality in *Pemphigus* aphids, with partial phylogeny of the genus. BMC Evolutionary Biology 2007, 7:185.
22. Coeur d’acier A, Cocuzza G, Jousselin E, Cavalieri V, Barbagallo S: Molecular phylogeny and systematics in the genus *Brachycaudus* (Homoptera: Aphididae): insights from a combined analysis of nuclear and mitochondrial genes. Zoologica Scripta 2008, 37: 175-193.
23. Kim H, Lee S: Molecular systematics of the genus *Megoura* (Hemiptera: Aphididae) using mitochondrial and nuclear DNA sequences. Molecules and Cells 2008a, 25: 510-522.
24. Kim H, Lee S: A molecular phylogeny of the tribe Aphidini (Isecta: Hemiptera: Aphididae) based on mitochondrial tRNA/COII, 12S/16S and the nuclear EF1alpha genes. Systematic Entomology 2008b, 33: 711-721.
25. Zang HC, Qiao GX: Molecular phylogeny of Fordini (Hemiptera: Aphididae: Pemphiginae) inferred from nuclear EF-1 alpha and mitochondrial gene COI. Bulletin of Entomological Research 2007, 97: 379-386.
26. Ortiz-Rivas B, Martinez-Torres D, Hidalgo NP: Molecular phylogeny of Iberian Fordini (Aphididae: Eriosomatinae): implications for the taxonomy of genera *Forda* and *Paracletus*. Systematic Entomology 2009, 34: 293-306.
27. Heie OE: Paleontology and phylogeny. In Aphids. Their biology, Natural Enemies and Control. Edited by Minks AK, Harrewijn P. Amsterdam: Elsevier; 1987: 367-391.
28. Wojciechowski W: Studies on the Systematic System of Aphids (Homoptera, Aphidinae). Katowice: Uniwersytet Slaski
29. Cook LG, Gullan PJ: The gall-inducing habit has evolved multiple times among the eriococcidae scale insects (Sternorrhyncha: Coccoidea: Eriococcidae). Biological Journal of the Linnean Society 2004, 83: 441-452.
30. Morse GE, Normark BB: A molecular phylogenetic study of armoured scale insects (Hemiptera: Diaspididae). Systematic Entomology 2006, 31: 338-349.
31. Andersen JC, Wu J, Gruwell ME, Gwiazdowski R, Santana SE, Feliciano NM, Morse GE, Normark BB: A phylogenetic analysis of armoured scale insects (Hemiptera: Diaspididae) based on nuclear, mitochondrial and endosymbiont sequences. Molecular Phylogenetics and Evolution, in press.
32. Hardy NB, Gullan PJ: *Opisthoscelis nigra* Froggatt (Eriococcidae) is an armoured scale *Maskellia nigra* (Froggatt) (Diaspididae). In: Proceedings of the XI International Symposium of Scale Insect Studies, Oeiras, Portugal, 24–27 September 2007. Edited by Branco M, Franco JC, Hodgson C. Lisbon: ISA Press; 2009: 59-62.
33. Ronquist F: Phylogeny, classification and evolution of the Cynipoidea. Zoologica Scripta 1999, 28: 139-164.
34. Buffington ML, Nylander JAA, Heraty JM: The phylogeny and evolution of Figitidae (Hymenoptera: Cynipoidea). Cladistics 2007, 23: 403-431.
35. Ács Z, Melika G, Pénzes Z, Pujade-Villar J, Stone G: The phylogenetic relationships between *Dryocosmus*, *Chilaspis* and allied genera of oak gallwasps (Hymenoptera, Cynipidae: Cynipini). Systematic Entomology 2007, 32: 70-80.
36. Nylander JAA, Ronquist F, Huelsenbeck JP, Nieves-Aldrey JL: Bayesian phylogenetic analysis of combined data. Systematic Biology 2004, 53: 47-67.
37. Ács Z, Challis RJ, Bihari P, Blaxter M, Hayward A, Melika G, Csóka G, Pénzes Z, Pujade-Villar J, Nieves-Aldrey J, Schönrogge K, Stone GN: Phylogney and DNA barcoding of inquiline oak gallwasps (Hymenoptera: Cynipidae) of the Western Palearctic. Molecular Phylogenetics and Evolution 2010, 55: 210-225.
38. Gauthier N, LaSalle J, Quicke DLJ, Godfray HCJ: Phylogeny of Eulophidae and recognition that Elasmidae are derived eulophids. Systematic Entomology 2000, 25: 521-539.
39. Manzari S, Polaszek A, Belshaw R, Quicke DL: Morphometric and molecular analysis of the *Encarsia inaron* species-group (Hymenoptera: Aphelinidae), parasitoids of whiteflies (Hemiptera: Aleyrodidae). Bulletin of Entomological Research 2002, 92: 165-176.
40. Chen Y, Xiao H, Fu J, Huang DW: A molecular phylogeny of urytomid wasps inferred from DNA sequence data of 28S, 18S, 16S, and COI genes. Molecular Phylogenetics and Evolution 2004, 31: 300-307.
41. Auger-Rozenberg M-A, Kerdelhue C, Magnoux E, Turgeon J, Rasplus J-Y, Roques A: Molecular phylogeny and evolution of host-plant use in conifer seed chalcids in the genus *Megastigmus* (Hymenoptera: Torymidae). Systematic Entomology 2005, 31: 47-64.
42. Gillespie JJ, Munro JB, Heraty JM, Yoder MJ, Owen AK, Carmichael AE: A secondary structure model of the 28S rRNA expansion segment D2 and D3 for chalcidoid wasps (Hymenoptera: Chalcidoidea). Molecular Biology and Evolution 2005, 22: 1593-1608.
43. Heraty J, Hawks D, Kostecki JS, Carmichael A: Phylogeny and behavior of the Gollumiellinae, a new subfamily of the ant-parasitic Eucharitidae (Hymenoptera: Chalcidoidea). Systematic Entomology 2005, 29: 544-559.
44. Lopez-Vaamonde C, Godfray HCJ, West SA, Hansson C, Cook JM: The evolution of host use and unusual reproductive strategies in *Achrysocharoides* parasitoid wasps. Journal of Evolutionary Biology 2005. 18: 1029-1041.
45. Taylor DB, Moon R, Gibson G, Szalanski A: Genetic and morphological comparisons of New and Old World populations of *Spalangia* species (Hymenoptera: Pteromalidae). Annual Review of the Entomological Society of America 2006, 99: 799-808.
46. Heraty JM, Woolley JB, Hopper KR, Hawks DL, Kim LW, Buffington M: Molecular phylogenetics and reproductive incompatibility in a complex of cryptic species of aphid parasitoids. Molecular Phylogenetics and Evolution 2007, 45: 480-493.
47. Schmidt S, Polaszek A: *Encarsia* or *Encarsiella*? – redefining generic limits based on morphological and molecular evidence (Hymenoptera, Apheliidae). Systematic Entomology 2007, 32: 81-94.
48. Gagné RJ: A catalog of the Cecidomyiidae (Diptera) of the world. Memoirs of the Entomological Society of Washington 2004, 25: 1-408.
49. Han H, Ro K, McPheron BA: Molecular phylogeny of the subfamily Tephritinae (Diptera: Tephritidae) based on mitochondrial 16S rDNA sequences. Molecules and Cells 2006, 22: 78-88.
50. Scheffer SJ, Winkler IS, Wiegmann BM: Phylogenetic relationships within the lean-mining flies (Diptera: Agromyzidae) inferred from sequence data from multiple genes. Molecular Phylogenetics and Evolution 2007, 42: 756-775.
51. Giblin-Davis RM, Center BJ, Davies KA, Purcell MF, Scheffer SJ, Taylor GS, Goolsby J, and Center TD: Histological comparisons of *Fergusobia*/*Fergusonina*-induced galls on different Myrtaceous hosts. Journal of Nematology 2004, 36: 249-262.
